# A patient with periumbilical hyperpigmentation and keratotic papules

**DOI:** 10.1016/j.jdcr.2025.05.003

**Published:** 2025-06-04

**Authors:** Mallory Hedden, Katelyn Dugan, Emily H. Smith, Alexander B. Aria

**Affiliations:** a Saint Louis University School of Medicine, St. Louis, Missouri; b SSM Health Department of Dermatology, Saint Louis University, St. Louis, Missouri

**Keywords:** perforating calcific elastosis, periumbilical

## Clinical images

### History

**Fig 1:**
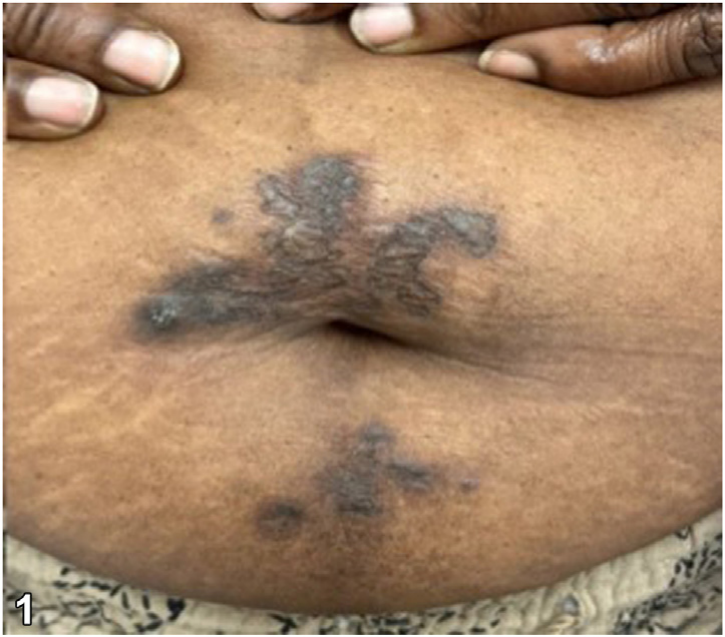
Perforating calcific elastosis. Well-demarcated hyperpigmented plaques with admixed focal keratotic papules and surrounding erythema in a periumbilical distribution.

**Fig 2:**
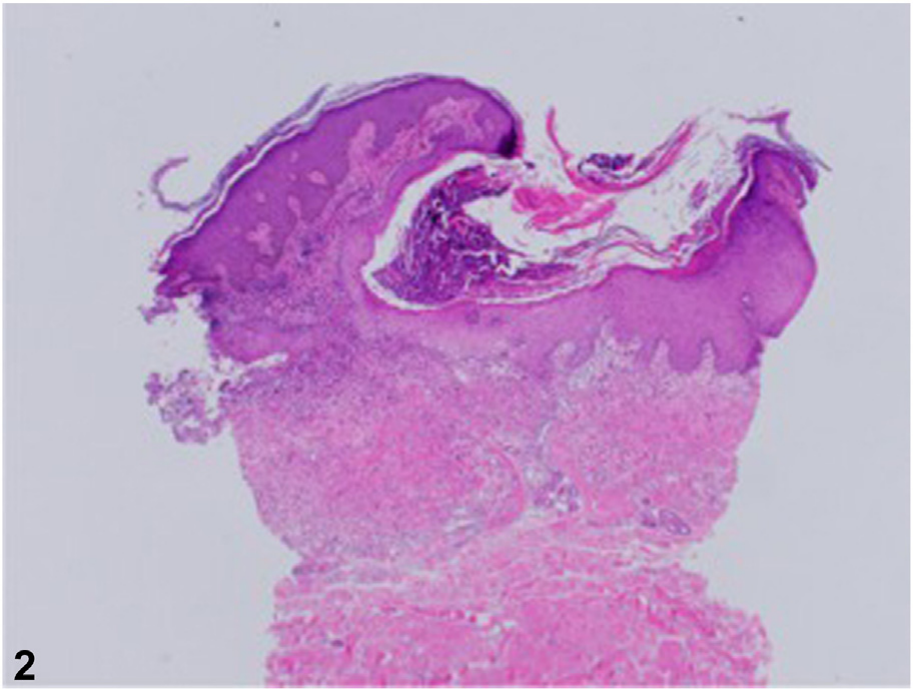
Hematoxylin and eosin stain demonstrating perforating calcific elastosis. Punch biopsy from the patient’s periumbilical region demonstrating short, curled, distorted collections of basophilic elastic fibers throughout the reticular dermis and extruding through a hyperplastic epidermis.

A 60-year-old multiparous African American female with a past medical history of hypertension, systemic lupus erythematosus, fibromyalgia, and osteoarthritis presented with a 3-year history of an abdominal rash. Associated symptoms included tenderness and worsening of the rash with friction. The patient had previously tried clobetasol 0.05% cream without improvement. She denied a family history of a similar abdominal rash. Examination revealed well-demarcated hyperpigmented plaques with admixed focal keratotic papules and surrounding erythema in a periumbilical distribution (Fig 1). Two punch biopsies were obtained from the right and inferior periumbilical abdomen. Hematoxylin and eosin staining showed short, curled, distorted collections of basophilic elastic fibers throughout the reticular dermis that extrude through a hyperplastic epidermis (Fig 2).

**Question: What is the most likely diagnosis?**

**A.** Perforating calcific elastosis
**B.** Localized hereditary pseudoxanthoma elasticum
**C.** Elastosis perforans serpiginosa
**D.** Calcinosis cutis
**E.** Reactive perforating collagenosis

**Answers:**

**A.** Perforating calcific elastosis—Correct. Perforating calcific elastosis, also known as periumbilical perforating pseudoxanthoma elasticum (PPPXE), is the most likely diagnosis in this middle-aged, multiparous patient given the clinical and histopathologic findings. Perforating calcific elastosis is an acquired perforating disorder caused by repetitive trauma that leads to degeneration and calcification of elastic fibers.
**B.** Localized hereditary pseudoxanthoma elasticum – Incorrect. Hereditary PXE is unlikely given this patient’s age of onset, absence of lesions on the flexural folds, lack of cardiovascular and ophthalmic involvement, and absence of family history of a similar rash.^1^
**C.** Elastosis perforans serpiginosa (EPS) – Incorrect. EPS can be differentiated from perforating PXE histologically by the presence of transepidermal elimination of unmineralized elastic fibers. EPS also primarily affects males and typically occurs in patients before age 30.^1^^,^^2^
**D.** Calcinosis cutis – Incorrect. Calcinosis cutis is characterized by dermal and subcutaneous nodules. Additionally, all connective tissue fibers are calcified in calcinosis cutis, while only the elastic fibers are calcified in perforating calcific elastosis.^1^
**E.** Reactive perforating collagenosis – Incorrect. Although RPC also presents with hyperkeratotic papules, it is characterized by transepithelial elimination of altered collagen, rather than elastic fibers, and is unlikely in this patient without a history of diabetes or renal disease.^3^

## Conflicts of interest

None disclosed.
